# Data analysis and presentation methods in umbrella reviews/overviews of reviews in health care: A cross-sectional study

**DOI:** 10.1017/rsm.2025.10040

**Published:** 2025-10-14

**Authors:** Cindy Stern, Jiaoli Li, Jennifer Stone, Hanan Khalil, Kim Sears, Romy Menghao Jia, Patraporn Bhatarasakoon, Edoardo Aromataris, Ritin Fernandez

**Affiliations:** 1 JBI, Faculty of Health and Medical Sciences, The University of Adelaide, Adelaide, SA, Australia; 2 Health Evidence Synthesis, Recommendations and Impact (HESRI), School of Public Health, Faculty of Health and Medical Science, The University of Adelaide, Adelaide, SA, Australia; 3 Center of Evidence-Based Education and Arts Therapies: A JBI Affiliated Group, Faculty of Education, Palacky University Olomouc, Olomouc, Czech Republic; 4 Department of Public Health, School of Psychology and Public Health, La Trobe University, Melbourne, VIC, Australia; 5 Queen’s Collaboration for Health Care Quality: A JBI Centre of Excellence, Queen’s University School of Nursing, Kingston, ON, Canada; 6 Department of Psychiatric Nursing, Faculty of Nursing, Chiang Mai University, Chiang Mai, Thailand; 7 Centre for Transformative Nursing, Midwifery, and Health Research: A JBI Centre of Excellence, School of Nursing and Midwifery, University of Newcastle, Callaghan, NSW, Australia

**Keywords:** data analysis, data presentation, methodology, overview, umbrella review

## Abstract

Umbrella reviews (URs) synthesize findings from multiple systematic reviews on a specific topic. Methodological approaches for analyzing and presenting UR results vary, and reviewers often adapt methods to align with research objectives. This study examined the characteristics of analysis and presentation methods used in healthcare-related URs. A systematic PubMed search identified URs published between 2023 and 2024. Inclusion criteria focused on healthcare URs using systematic reviews as the unit of analysis. A random sample of 100 eligible URs was included. A customized, piloted data extraction form was used to collect bibliographic, conduct, and reporting data independently. Descriptive analysis and narrative synthesis summarized findings. The most common terminology for eligible studies was “umbrella reviews” (65%) or “overviews” (30%). Question frameworks included PICO (43%) and PICOS (14%), with quantitative systematic reviews included in most URs (98%), and 68% including randomized controlled trials. The most frequent methodological guidance source was Cochrane (32%). Data analysis commonly used narrative synthesis and meta-analysis, with Stata, RevMan, and GRADEPro GDT employed for presentation. Information about study overlap and certainty assessment was rarely reported.Variation exists in how data are analyzed and presented in URs, with key elements often omitted. These findings highlight the need for clearer methodological guidance to enhance consistency and reporting in future URs.

## Highlights

### What is already known?

- Umbrella reviews (URs) aim to integrate and analyze findings from multiple systematic reviews on a given topic.
- URs are a popular type of evidence synthesis.
- Limited understanding of the methodologies used to analyze and present findings of URs exist.

### What is new?

- This cross-sectional study of a sample of 100 URs showed a strong emphasis on synthesizing quantitative data with limited examples of URs containing qualitative data located.
- Software used in analysis and presentation is often not reported.
- Many URs do not address primary study overlap nor assess certainty of the evidence.

### Potential impact for Research Synthesis Methods readers

- Reviewers conducting URs should assess primary study overlap, assess certainty of the evidence, and report software used for analysis and presentation of data.

## 1 Introduction

An umbrella review (UR), also known as an “overview of reviews (ORs),” “systematic reviews,” or “meta-review,” aims to integrate and analyze the findings from multiple systematic reviews (SRs) on a given topic.^1^ This distinction between an UR and a SR lies in the unit of analysis, with SRs focused at the primary study level, while URs are focused at the SR or other secondary research synthesis level.

Umbrella reviews are becoming popular given the explosion of SRs being published annually^2^^,^ ^3^ and the increasing need to synthesize their findings. URs provide a higher level summary of the body of knowledge on a particular topic and also identifies similarities and differences in the evidence base.^1^ They can have a much broader scope depending on the type of question/s posed and play a crucial role in informing clinical decision making, policy development, and evidence-based practice.

In terms of conduct, the steps for an UR are similar to that of a SR, however, there are some nuances that relate to their unit of analysis. Some of these pertain to how data are analyzed and presented. Several methods for analyzing and presenting the results of URs have been proposed. For example, JBI (formally the Joanne Briggs Institute) methodological guidance for quantitative URs does not currently recommend reanalysis of results of SRs using meta-analysis, instead encouraging summary tables with sufficient descriptive detail^4^ and a narrative report. In contrast, Cochrane methodological guidance^5^ recommends two methods of data analysis (1) summarizing data and (2) reanalyzing outcome data in a way that differs from the original analyses conducted in the SRs (e.g., to analyze specific populations or subgroups).

In contrast, attempts to establish methodological guidance for qualitative evidence at the UR level are in their infancy. Although methods to analyze qualitative evidence such as mega-aggregation^6^ and mega-ethnography^7^ are recommended for answering different review questions, guidance on presentation is more limited—with JBI again recommending tabular form of synthesized findings.^4^

Other important issues related to the analysis of data in URs include assessing primary study overlap within the included SRs,^8^ assessing the methodological quality of SRs, assessing the certainty/confidence of evidence of SRs, and reporting requirements with various methods and standards available.^9^^–^ ^12^ Despite the increasing prevalence of URs in health research, there is limited understanding of the specific methodologies used for analysis and presentation and their impact on the interpretation of findings.

The JBI Umbrella Review Methodology Group is an international group of methodologists and researchers who report to the JBI Scientific Committee^13^ and is tasked with providing up-to-date guidance and materials on JBI’s approach to URs.^4^ The group is currently undertaking a major update of its guidance with the first piece of work focused on data analysis and presentation methods. To inform methodological guidance, further investigation into the different methods and approaches used by UR authors is useful. This study aimed to examine the characteristics of analysis and presentation approaches used in a sample of URs related to health care. By identifying common trends and methodological choices, this research seeks to determine the transparency, consistency, and utility of URs in the healthcare domain.

## 2 Research question

What are the characteristics of analysis and presentation methods used in URs informing health care?

## 3 Method

The study was undertaken in accordance with a protocol registered in Open Science Framework (OSF) (https://osf.io/a9eu6). It followed the methodology of epidemiological studies, where a random sample of studies was selected to address the research question.^14^ The steps undertaken included defining inclusion and exclusion criteria, developing a search strategy to identify eligible URs, screening and selection of search results, and data extraction and analysis.

### 3.1 Search strategy

The search for URs was conducted in PubMed (JL) with a time frame between March 5, 2023 and March 5, 2024. PubMed was selected as the sole database to answer the research question because it provides extensive coverage of biomedical literature relevant to this study, including the majority of key healthcare journals. This 1-year time period was chosen to ensure the feasibility and currency of the project. As there are no subject headings, subsets, or search filters for URs currently available in PubMed, the search strategy developed by Pieper and colleagues^8^ was adapted during consultation with a librarian (RJ) (see Appendix A).

### 3.2 Inclusion and exclusion criteria

Umbrella reviews with a focus on health were included. Reviews were restricted to the English language due to team capacity. For the purpose of this project, the focus was exclusively on URs that contained SRs or meta-analyses as their unit of analysis; reviews including scoping reviews, literature reviews, and primary studies in addition to SRs were excluded. Methodological reviews were also excluded.

### 3.3 Sample size

This cross-sectional study aimed to include 100 URs which were randomly selected from the search results using a random number generator.^15^ The sample size was agreed upon by the project team as it was considered sufficient to address the research question while remaining feasible with the available resources.

### 3.4 Screening and selection

Search results were imported into Excel and screened at title and abstract by two independent screeners (JL and JD) with any disagreements resolved by a third reviewer (CS). A random number generator was then used to select 100 records which were then imported into Covidence (an online platform used to conduct systematic reviews). It was anticipated that some records may not meet inclusion criteria at full-text review to get to the agreed sample size so an additional 20 records were randomly selected and set aside to use following full-text review if required. Full-text screening was undertaken by five screeners (CS, RF, JL, PB, and HK) and a pilot on five records was conducted to ensure consistency between screeners.

### 3.5 Data extraction and analysis

A data extraction form (Appendix B) was created and revised following group discussion. A pilot test was undertaken to assess the robustness of the data extraction tool, and relevant changes were subsequently made. Eight URs were included in the pilot phase, two more than originally specified in the review protocol. This modification was introduced to accommodate additional study team members, with each pair piloting two different papers. Data focusing on bibliographical information, descriptive characteristics of the URs (e.g., methodological and reporting guidance followed, critical appraisal tool used), and analysis and presentation methods (e.g., certainty of evidence method used and software used) were extracted. While the protocol specified that all extractions would be undertaken in duplicate, it was decided that extractions would be conducted by one author and a sample of extractions validated by another author (JL or CS) due to the type of data being extracted. Covidence was employed as a tool to manage the extraction process. In total, 20 extractions were verified representing 20% of included reviews. Any disagreements were addressed by discussion.

A descriptive analysis of the results, including tabular presentation and narrative, was conducted. Where appropriate, frequency distributions were presented, with percentages calculated and rounded to the nearest whole number. The mean, standard deviation, and median were calculated for the number or reviews included in the URs.

## 4 Results

### 4.1 Study inclusion

**Figure 1:**
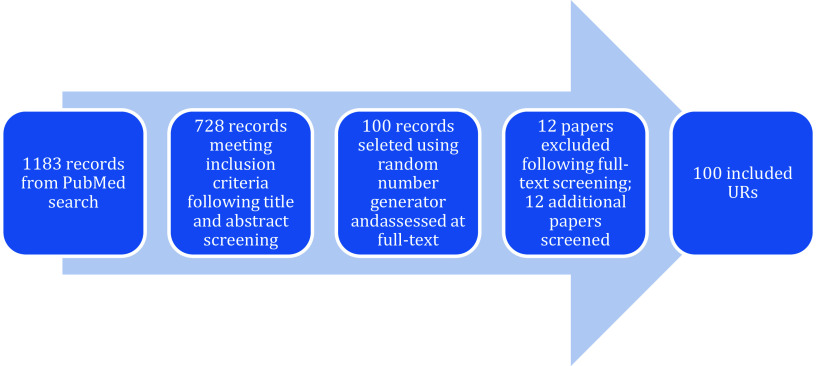
Study identification and inclusion flow chart.

The PubMed search was conducted on April 30, 2024, resulting in 1183 records identified and title and abstracts screened for eligibility. Following screening, 728 records remained and using the random number generator,^15^ 100 records were selected and screened at full text. Of those, 12 were excluded (11 did not exclusively include SRs or meta-analyses as their unit of analysis and one was not an UR), so a further 12 records were selected from the additional set of 20 records (described previously). One hundred URs were included in the study (see Figure 1). A table of UR characteristics is presented in Supplementary Material 1, a summary table of the results is presented in Supplementary Material 2, and the citation details of the reviews is included in Supplementary Material 3.

### 4.2 Review nomenclature

Seventy-one umbrella reviews were published in 2023 and 29 in 2024. Topics were diverse across the health spectrum. Most included reviews referred to themselves as an “umbrella review” (*n* = 65, 65%); this was followed by an “overview” (*n* = 30, 30%). Seven reviews (7%) used variations of the term “systematic review” (e.g., systematic review of systematic reviews, systematic review of reviews, systematic review of meta-analyses), and two reviews used the term “rapid review” (e.g., rapid review of reviews, rapid review of systematic reviews). Some reviews used multiple terms within the article.

### 4.3 Framework for question formulation

The majority of URs (*n* = 66, 66%) referred to either a protocol being published or publicly available or the UR being registered. Over half of the included URs utilized a PICO format (*P*opulation, *I*ntervention, *C*omparator, *O*utcome) for their question development and/or eligibility criteria (*n* = 43 [43%] PICO, *n* = 14 [14%] PICOS [Study design or Setting], *n* = 1 [1%] PICOTS [Time]). Eight percent of URs (*n* = 8) used a version of the Population, Exposure, Comparator, Outcome (PECO) format (PECO [*n* = 1, 1%], PECOS [Study design] [*n* = 1, 1%], PEO [*n* = 6, 6%]), while four URs (4%) used PICo (Population, Interest, Context). Two URs (2%) used PCC (Population, Concept, Context) and one UR (1%) used SPIDER (Sample, Phenomenon of Interest, Design, Evaluation, Research Type). Population, Outcomes, and Comparator were used once (1%), as were Population and Phenomena of interest (1%). A quarter of URs did not explicitly specify the framework used (*n* = 25, 25%).

### 4.4 Review composition

The number of SRs included in 99 URs ranged from 4 to 175 with a mean of 24.3 (standard deviation [SD] = 24.5) and a median of 16. One UR did not report the number of SRs but instead provided the total number of RCTs included in all SRs in the UR.

The majority of the URs included quantitative SRs (*n* = 98, 98%), either exclusively or alongside qualitative or mixed method SRs. Seven percent of URs (*n* = 7) also included meta-analyses, while one (1%) also included a network meta-analysis.

Of the URs that contained qualitative evidence, most were from mixed methods SRs (*n* = 7, 7%) with only 2 URs located that exclusively included qualitative SRs (*n* = 2, 2%). Additionally, one UR (*n* = 1) included narrative reviews (*n* = 1, 1%).

Considering the designs of primary studies included in the SRs, the most frequently mentioned were randomized controlled trials (RCTs) in 68% of URs (*n* = 68). This was followed by cohort studies and case–control studies, included in 36 (36%) and 26 (26%) of URs, respectively. Additionally, quasi-experimental studies and cross-sectional studies were reported in 24% (*n* = 24) and 20% (*n* = 20) of the URs, respectively. Other designs included in the SRs were case series/reports (*n* = 12, 12%), prevalence studies (*n* = 3, 3%), and mixed methods studies (*n* = 4, 4%). Six URs (6%) included qualitative studies. Notably, 12 URs (12%) did not mention what study designs were included, representing a lack of methodological detail.

### 4.5 Methodological and reporting guidance

In 55 URs (55%), there was no mention of methodological guidance followed. Cochrane methodological guidance^5^ was referred to in 32 URs (32%), while 14 used JBI guidance^4^ (14%). One UR cited both Cochrane and JBI methodological guidance (1%), while three reviews cited Cochrane guidance and other sources (3%).

In terms of reporting guidance, the majority of reviews stated they followed the *P*referred *R*eporting *I*tems for *S*ystematic Reviews and *M*eta-*A*nalyses^16^ (PRISMA) reporting standards (*n* = 73, 73%), followed by the *P*referred *R*eporting *I*tems for *O*verviews of *R*eviews^9^ (PRIOR) (*n* = 15, 15%) and *P*referred *R*eporting *I*tems for *O*verviews of Systematic Reviews including *harm* checklist^17^ (PRIO-harms) (*n* = 3, 3%). Other guidance mentioned included *En*hancing *T*ransparency in *Re*porting the Synthesis of *Q*ualitative Research^18^ (ENTREQ), the PRISMA extension for *d*iagnostic *t*est *a*ccuracy^19^ (PRISMA-DTA), *B*rief *R*eview *C*hecklist (BRC), and the PRISMA extensions for *sc*oping *r*eviews^20^ (PRISMA-ScR), with one review, respectively (1% each). Sixteen percent of reviews (*n* = 16) made no mention of reporting guidance.

### 4.6 Primary study overlap

Primary study overlap refers to two of more systematic reviews or meta-analyses included in a UR that share one or more of the same primary studies. Study overlap is a unique methodological challenge in URs and can impact validity and reliability. Primary study overlap was reported in 48 included URs (48%); 21 URs used corrected covered area calculations^8^ (21%), 16 provided a narrative description (16%), and 15 presented a tabular display (15%). The Graphical Representation of Overlap for OVErviews^21^ (GROOVE) was mentioned in two URs and 14 URs (14%) included multiple formats. Of those URs where the overlap of the primary studies was considered and where multiple outcomes were reported, 13 reviews (13%) reported the overlap for individual outcomes.

### 4.7 Critical appraisal

Critical appraisal is the process of assessing the methodological quality of included systematic reviews or meta-analyses included in a UR and is considered an essential step in the review process. Almost all URs (*n* = 97, 97%) undertook critical appraisal of the included SRs. The most common instrument used was AMSTAR-2 (A MeaSurement Tool to Assess systematic Reviews)^11^ (*n* = 69, 69%), followed by the JBI tool^4^ (*n* = 10, 10%), ROBIS^12^ (*n* = 10, 10%), and the original AMSTAR^22^ (*n* = 8, 8%). Other URs referred to various tools such as but not limited to: SANRA (Scale for the Assessment of Narrative Review Articles)^23^ criteria for narrative reviews, the Health Evidence checklist for systematic reviews,^24^ and the Cochrane Risk of Bias tool^25^ (*n* = 1, 1%, respectively).

Only four URs (4%) excluded SRs based on methodology quality. The threshold/criteria used for this decision varied, but related to excluding reviews that were considered low or very low methodological quality.

Of them, three used the JBI tool, while one used AMSTAR-2. For the three URs using the JBI tool, the following details were provided:

- One UR excluded low-quality reviews with low determined if the score was below 40%^26^
- One UR excluded two papers whose quality level was lower than 40%^26^
- One UR planned to exclude studies with a minimum score of 4^27^

The UR that used AMSTAR-2 stated that “3 were rejected due to very low confidence according to AMSTAR 2.”^28^

### 4.8 Data analysis

All URs containing quantitative data analyzed data narratively. Of the 98 URs (98%) that analyzed quantitative data only, 30 (30%) included meta-analysis, 27 (27%) utilized basic descriptive analysis, and 3 (3%) used some form of data categorization.

Of the seven URs (7%) that included qualitative data (either from qualitative reviews or mixed methods reviews), seven URs (7%) analyzed data narratively, three included a method of qualitative synthesis (3%), and one each reported basic descriptive analysis (1%) and a process of categorization (1%).

Of the 100, 27 included URs (27%) referred to using software to analyze or present data. Tools used included Stata (*n* = 12), GRADEPro GDT (*n* = 5), MS Excel (*n* = 5), RevMan (*n* = 5), R (*n* = 3), Meta-umbrella (*n* = 2), and Comprehensive Meta-analysis (*n* = 2). Other software such as NVIVO, SPSS, Cytoscape, MetaXl, VOS Viewer, and STATISTICA were each mentioned once (*n* = 1).

### 4.9 Data presentation

While all URs including quantitative data provided narrative to describe their results, eight (8%) exclusively used text for presentation. To support the presentation of the results, the most popular method used was tabular display (*n* = 76, 78%), followed by forest plots (*n* = 29, 30%), and Summary of Findings tables (*n* = 18, 18%). Other approaches included figures (*n* = 5, 5%), the JBI stop light table (*n* = 3, 3%), bar charts (*n* = 3, 3%), and one each used line graphs, heat maps, evidence maps, funnel plots, and radar plots (1%). Over a third of URs used multiple presentation formats in addition to text (*n* = 41, 41%).

Of the eight URs containing qualitative data, five (63%) utilized tabular display to present their results, two used Summary of Findings (25%), and two used figures (25%).

The majority of URs presented between one and five tables or figures. A small number of URs (*n* = 2, 2%) reported no tables or figures, while 7% (*n* = 7) included only one. A larger portion of URs (*n* = 14, 14%) included two tables or figures, and 19% (*n* = 19) included three. Most URs used between four (*n* = 17) and five (*n* = 20) tables or figures (37% combined), with nine (9%) reporting six tables or figures. Including more than six tables or figures was rare, with only seven URs (7%) using seven or more, including one (1%) UR with 32 tables or figures.

### 4.10 Data presented in supplementary materials

Of the 50 (50%) URs that included Supplementary Materials, 23 (46%) provided data directly related to the analysis of results. Among these, 16 (32%) were associated with the main analyses, encompassing meta-analyses of primary data (*n* = 3, 6%), unspecified main analyses (*n* = 1, 2%), robust variance estimation (RVE) techniques (*n* = 1, 2%), statistical test results and reported associations (*n* = 2, 4%), subgroup analyses (*n* = 2, 4%), and narrative synthesis (*n* = 2, 4%). Presentation of results was also common, with additional forest plots (*n* = 7, 14%), funnel plots (*n* = 1, 2%), and other visual outputs such as figures, charts, or detailed tables (*n* = 5, 10%). Additionally, seven (14%) URs included assessments of overlap within their Supplementary Materials, while five (10%) provided data related to supplementary analyses, such as sensitivity analyses (*n* = 2, 4%) and other forms of additional analysis not featured in the main text (*n* = 3, 6%).

### 4.11 Certainty assessment

Certainty assessment (confidence assessment is the term often used for qualitative research) refers to determining how trustworthy and applicable a body of evidence is. Just over half of the URs did not assess the certainty of the evidence (*n* = 52, 52%). Of those that did assess certainty (48%), most used the Grading of Recommendations Assessment, Development, and Evaluation^29^ (GRADE) approach (*n* = 39, 39%). Other approaches included Confidence in the Evidence from Reviews of Qualitative research^30^ (CerQual) (*n* = 4, 4%) and NutriGrade^31^ (*n* = 2, 2%). Eight URs (8%) referred to using other individual methods with some referencing other documents/criteria (e.g., the SIGN Scale), while others appeared to create their own criteria for assessment (e.g., The overall confidence [high, moderate, low, and critically low] was graded as follows—high: no or one noncritical weakness; moderate: more than one noncritical weakness; low: one critical flaw with or without noncritical weaknesses; and critically low: more than one critical flaw with or without noncritical weaknesses).

## 5 Discussion

The application and use of umbrella/overview methods by review authors are diverse with many details related to key UR elements often not reported. The nomenclature used to describe the review methodology, “umbrella review” was by far the most common term used. This term reflects the growing recognition of URs as a distinct and important methodology within the evidence synthesis family.^13^

Our dataset predominantly consisted of URs that included quantitative evidence, which was evident when looking at the designs of the primary studies (RCTs, cohort studies and case–control studies being the most popular), and the use of a PICO framework for formulating research questions. For URs containing quantitative data, some authors undertook meta-analyses; Cochrane does advocate for further analysis if reanalyzing data in a different way, however, it was not in the scope of this project to investigate the appropriateness of the analysis and further research is required. Few URs included qualitative (or mixed methods) evidence, suggesting a trend in statistical synthesis for evidence-based decision making. As URs evolve, integrating qualitative research to capture contextual and experiential insights will enhance the applicability of findings.

The use of narrative synthesis, meta-analysis, and basic descriptive analysis in URs highlights the importance of clear and structured methods for synthesizing quantitative evidence. Most URs analyzed data narratively and presented it in tabular form, which aligns with JBI guidance^4^ and Cochrane guidance.^5^ The prevalence of tabular displays, forest plots, and summary of findings tables that align with GRADE methodology^32^ emphasizes the need for effective presentation of complex data in ways that enhance translation. There was, however, a small portion presenting results as text only. For qualitative and mixed methods data, the predominant use of narrative synthesis and categorization reflects standard practices in qualitative SRs. Importantly, less than a third of reviews mentioned the software used for analysis and presentation of results, and Stata was the most commonly cited tool. While software tools are widely used, there is still a lack of consistent reporting on their application in URs.

More than half of the reviews did not address primary study overlap, and those that did used different approaches, such as the corrected covered area or citation matrices. Primary study overlap is a distinct challenge in URs, as it can lead to the overrepresentation of certain studies, thereby distorting effect estimates and reducing the reliability of synthesized findings.^33^ Ways to manage study overlap have been established (including frameworks such as Methods for Overviews of Reviews [MOoR],^34^ which details the stages of a UR where overlap can be managed and outlines methods to do so), however, methods are still developing.^35^^,^ ^36^ More than half of the included URs in this study made no mention of study overlap highlighting a critical gap in methodological transparency. For those that did consider overlap, most did not report it for individual outcomes indicating that further standardization in how overlap is reported may be needed. Future methodological advancements should focus on developing standardized reporting frameworks and best-practice guidelines to ensure the reliability of data synthesis in URs.

Over half of the URs did not assess the certainty of the evidence, despite this being a key component of SR methodology as outlined in both the PRIOR statement^9^ and the PRISMA statement.^16^ For those that did assess certainty, the GRADE framework was most commonly used, however, it is important to note that at present there is no formal guidance from GRADE on how to assess certainty in URs.^37^ This finding indicates a gap in methodological guidance that could benefit from further development to ensure that URs consistently incorporate robust certainty assessments.

Over half of the included URs did not explicitly state which methodological conduct guidance they followed, but more than three quarters reported adhering to reporting standards, such as PRISMA.^16^ The lack of explicit guidance adherence for methodological conduct suggests ongoing variability in research practices, evident in this study, potentially impacting the reliability and comparability of findings. This variability underscores the need for clearer implementation of methodological standards to enhance consistency and credibility in UR conduct and reporting.^16^

The substantial variability in methodologies, reporting practices, and analytical approaches highlight the need for clear, standardized guidance for researchers conducting URs. Establishing comprehensive methodological frameworks will enhance consistency, transparency, and the overall quality of evidence synthesis. The findings of this study provide a crucial foundation for identifying key gaps and areas for improvement, guiding the development of standardized protocols and best practices. By addressing these inconsistencies, future research can ensure more reliable and comparable results, ultimately strengthening the impact of URs in evidence-based decision making.

### 5.1 Study limitations

This study only included URs in English and there may be URs available in languages other than English that meet our inclusion criteria and thus were missed. The study was limited exclusively to URs related to health care with only one database searched which means there may be characteristics used across other disciplines that were not identified. We restricted inclusion to URs that exclusively contained SRs or meta-analyses as their unit of analysis, and it is important to note that the sample size may not represent the diversity of URs across different disciplines in health. Not all extractions were undertaken in duplicate which could have introduced errors in data extraction. Finally, the study did not explore the influence of potential conflicts of interest or funding sources, which could affect the design and reporting of URs.

## 6 Conclusion

This is the first in a series of methodological projects being undertaken to inform the update of the JBI UR methodological guidance. This study provides valuable insights into the methodologies, methods, and characteristics of URs within health care. The findings reveal a strong emphasis on synthesizing quantitative data, with a growing interest in integrating qualitative and mixed methods evidence. Adherence to established methodological frameworks, such as PRISMA for reporting and Cochrane and JBI for conduct, is generally high, though variability in reporting practices remains. The study also highlights many elements of the methodology that are not being included in URs with key areas for improvement, including standardized reporting of study overlap and the need for more consistency in certainty assessments. These results underscore the evolving nature of URs and the need for further refinement in their methodological practices.

## Supporting information

Stern et al. supplementary material 1 Stern et al. supplementary material

Stern et al. supplementary material 2 Stern et al. supplementary material

## Data Availability

The authors confirm that the data supporting the findings of this study are available within the article. The raw data are provided as Supplementary Material.

## Supplementary material

To view supplementary material for this article, please visit http://doi.org/10.1017/rsm.2025.10040.

## Appendix A: Search strategy

### A.1 PubMed

(((((((((((((((((((umbrella review[Title]) OR (meta review[Title])) OR (metareview[Title])) OR ((overview[Title]) AND ((reviews[Title/Abstract]) NOT (reviews[Title])))) OR ((overview[Title]) AND (reviews[Title]))) OR ((overview[Title]) AND ((meta analyses[Title/Abstract]) NOT (meta analyses[Title])))) OR ((overview[Title]) AND ((metaanalyses[Title/Abstract]) NOT (metaanalyses[Title])))) OR ((overview[Title]) AND (meta analyses[Title]))) OR ((overview[Title]) AND (metaanalyses[Title]))) OR (((meta analyses[Title/Abstract]) NOT (meta analyses[Title])) AND (review[Title]))) OR (((metaanalyses[Title/Abstract]) NOT (metaanalyses[Title])) AND (review[Title]))) OR (((meta analyses[Title])) AND (review[Title]))) OR ((metaanalyses[Title]) AND (review[Title]))) OR ((review[Title]) AND (reviews[Title]))) OR ((metaanalyses[Title]) AND (synthesis[Title]))) OR ((meta analyses[Title]) AND (synthesis[Title]))) OR ((reviews[Title]) AND (synthesis[Title]))) OR ((metaanalyses[Title]) AND (summary[Title]))) OR ((meta analyses[Title]) AND (summary[Title]))) OR ((reviews[Title]) AND (summary[Title])) Filters: from 2023/3/5–2024/3/5.

## Appendix B: Data extraction form

**Table 1:**

Field	Explanation
Title (free-text response)	Verbatim from article
Nomenclature (free-text response)	A review may nomenclate itself by various terms like umbrella review, overview, or meta-review… For example: Title: A Call for Improving Research on Pain Neuroscience Education and Chronic Pain: An Overview of Systematic Reviews. Nomenclature: An Overview of Systematic Reviews.
Authors, publication year (free-text response)	The full name of the first author; year of publication
Protocol available (Pre-set option—choose 1) Yes No	If review mentions a protocol was registered, or refers to a published or publicly available protocol, or available upon request, this should be marked as “yes.”
Review question (free-text response)	Verbatim from article. If multiple questions, include all.
Methodological guidance followed (tick all that apply) Cochrane methodological guidance JBI methodological guidance COSMOS-E Other(list) Not mentioned	COSMOS-E: Guidance on conducting systematic reviews and meta-analyses of observational studies of etiology
Reporting guidance followed (tick all that apply) PRIOR PRISMA ENTREQ Other(list) Not mentioned	PRIOR: Preferred Reporting Items for Overviews of Reviews (PRIOR) guideline The Preferred Reporting Items for Systematic reviews and Meta-Analyses (PRISMA) statement The Enhancing transparency in reporting the synthesis of qualitative research (ENTREQ) statement
Question Framework/Mnemonic used (free-text response)	e.g., PICO, PICo… The review may not specifically state one but we can tell from the eligibility criteria, etc.
What types of systematic reviews (SRs) were included? (Pre-set option—choose 1) Effectiveness Qualitative Mixed method (quant + qual) Prevalence and incidence Economic evidence Etiology and risk Diagnostic test accuracy Measurement properties Text and opinion/textual Other (list)	Identified by the umbrella review question and analyzed data.
How many SRs were included in data synthesis stage? (free-text response)	SRs may be excluded after critical appraisal. Only number of SRs included in the data synthesis should be presented.
What *types of primary studies* were included in those included SRs? (tick all that apply) RCT Quasi-experimental studies Cohort studies Case–control studies Cross-sectional studies Case series/case report studies Qualitative studies Mixed methods Other (list) Not mentioned	Usually mentioned in the Method.
How did the UR consider *the overlap of the primary studies* within SRs? (tick all that apply) Corrected covered area calculation Tabular form Narrative description Other(list) Not mentioned	Article may show the overlap frequency of the primary studies by a table, or they calculated the “corrected covered area.”
If overlap of primary studies was considered in the UR and multiple outcomes were reported, was the overlap reported for individual outcomes? (Pre-set option—choose 1) Yes No Not applicable	
What critical appraisal tool was used in the UR? (select all that apply) and identify the number of times each tool was used. AMSTAR AMSTAR 2 ROBIS JBI critical appraisal tool for systematic reviews and research syntheses Other (list) Tool not mentioned Critical appraisal not undertaken	AMSTAR: A MeaSurement Tool to Assess systematic Reviews AMSTAR 2: A MeaSurement Tool to Assess systematic Reviews 2 ROBIS: the Risk of Bias in Systematic Reviews
Did the UR *exclude* any reviews based on methodological quality? Yes If yes please specify the threshold/criteria used (free text) No	If the article did not mention that SRs were excluded for poor methodological quality, it should be marked as “No.”
How did the authors analyze the data of existing systematic reviews? (tick all that apply) Quantitative Narratively Basic descriptive statistics Meta-analysis Other statistical approaches (please list)	
Qualitative Narratively Basic descriptive statistics Qualitative synthesis (please list) Other approaches (please list)	
How did the UR present the results? (tick all that apply and for each, include the number of figures/tables included in the UR) Quantitative JBI “stop-light” figure “Summary of findings” Tabular display Narrative text only Forest plot Horseshoe plot Harvest plot Other (list) Qualitative JBI “stop-light” figure “Summary of findings” Tabular display Narrative text only Other (list)	e.g., forest plot e.g., horseshoe plot e.g., harvest plot e.g., JBI “stop-light” table
How did the authors assess *the certainty of evidence*? (tick all that apply) GRADE CerQual ConQual Other(list) Certainty not assessed	
What Software was used for data analysis? (free-text response)	
What Software was used for presentation of data? (free-text response)	
Are additional data presented in Supplementary Materials? Yes No
If yes, What data were provided in the Supplementary Materials? (free-text response) How was it presented? Quantitative (tick all that apply) JBI “stop-light” figure “Summary of findings” Tabular display Narrative text only Forest plot Horseshoe plot Harvest plot Other (list) Qualitative JBI “stop-light” figure “Summary of findings” Tabular display Narrative text only Other (list)	
