# Supplementary material for: Data analysis and presentation methods in umbrella reviews/overviews of reviews in health care: A cross-sectional study
Source: Res Synth Methods. 2025 Oct 14;17(1):210–24. doi: 10.1017/rsm.2025.10040 (PMC12823197; doi:10.1017/rsm.2025.10040)
Supplement: Stern et al. supplementary material 2 — Stern et al. supplementary material [file S1759287925100409sup002.docx]

Supplementary file 1: Table of included reviews

| **Author/Year** | **Review Question** | **Number of SRs included in synthesis** | | **Types of SRs included** | | **Types of primary studies included** | |
| --- | --- | --- | --- | --- | --- | --- | --- |
| Zhou 2024 | This study used the AMSTAR 2 tool and the GRADE tool to evaluate the methodological and evidence quality of published systematic reviews (SRs)/meta-analyses (MA) and to grade and summarize the quality of the evidence to provide more comprehensive and reliable evidence-based evidence for Extracorporeal shockwave therapy for Knee osteoarthritis | | 8 | | Quantitative reviews | | Randomized controlled trials |
| Carregaro 2023 | Is the inactivated quadrivalent influenza vaccine more immunogenic and safer compared to the trivalent vaccine? | | 5 | | Quantitative reviews | | Randomized controlled trials |
| Zhang 2024 | To comprehensively collect, evaluate and synthesize the evidence from SRs/MAs in this field | | 7 | | Quantitative reviews | | Randomized controlled trials |
| Valente 2024 | To conduct an umbrella review to summarize all available systematic reviews and meta-analyses investigating the efficacy and safety of valerian concerning to sleep problems, namely insomnia. | | 8 | | Quantitative reviews | | Randomized controlled trials; Quasi-experimental studies; Cohort studies; Case control studies; Cross sectional studies; Case series/case reports |
| Tesfaye 2024 | To provide a comprehensive summary of the available evidence on the extent and predictors of medication nonadherence, the outcomes associated with nonadherence, and the interventions implemented across the health care continuum to improve medication adherence and associated outcomes in patients with different stages of Chronic Kidney disease | | 37 | | Quantitative reviews; Qualitative reviews; Mixed methods (quant+ qual) reviews; Other: meta-analyses | | Randomized controlled trials; Quasi-experimental studies; Cohort studies; Cross sectional studies; Case series/case reports; Qualitative studies |
| Goh 2024 | The aim of this overview of reviews is to appraise and synthesise evidence from systematic reviews, that have investigated the impact of self-management interventions for people with epilepsy on clinical outcomes, including health-related quality of life, self-efficacy, medication compliance, seizure status, and psychosocial well-being | | 12 | | Quantitative reviews; Qualitative reviews; Mixed methods (quant+ qual) reviews | | Randomized controlled trials; Qualitative studies; Mixed methods studies |
| Berthelsen 2024 | How are the content and timeframe of the interventions comprising elements from the Transitional Care Model described? What patient outcomes are improved using elements of the  Transitional Care Model? | | 5 | | Quantitative reviews | | Randomized controlled trials; Quasi-experimental studies; Case series/case reports |
| Shafiee 2023 | To summarize and critically appraise the body of evidence on conservative management of the complex regional pain syndrome and to provide an overall picture of the current state of the literature | | 9 | | Quantitative reviews | | Randomized controlled trials |
| Peitsidis 2023 | Evaluating the use of aromatase inhibitors in the clinical management of endometriosis-related symptoms. In addition, we performed a systematic review of the narrative reviews in the international literature | | 24 | | Quantitative reviews; Other: Narrative reviews | | Randomized controlled trials; Quasi-experimental studies; Cohort studies; Case series/case reports |
| Shi 2023 | To critically assess and scientifically document the quality of SRs/ MAs on acupuncture for chemotherapy-induced peripheral neuropathy | | 9 | | Quantitative reviews; Other: meta-analyses | | Randomized controlled trials |
| Mazumder 2024 | To provide a comprehensive evaluation and synthesis of existing epidemiological evidence between exposure to air pollution (any type of air pollutants) and maternal health outcomes (any physical or mental health outcomes of mothers during pregnancy and postpartum period) | | 20 | | Quantitative reviews | | Cohort studies; Case control studies; Cross sectional studies |
| Wang 2023 | The association between ultra-processed foods and the risk of hypertension in various populations | | 7 | | Quantitative reviews | | Cohort studies; Cross sectional studies |
| Viderman 2023 | This umbrella review aims to estimate the prevalence of pain among patients with different chronic diseases/conditions. | | 25 | | Quantitative reviews | | Prevalence studies |
| Sananès 2023 | To provide an overview of the evidence found in Cochrane reviews concerning the impact of interventions during pregnancy, labour and the postpartum period on the prevention of urinary and faecal incontinence | | 9 | | Quantitative reviews | | Randomized controlled trials; Quasi-experimental studies |
| Kaldal 2023 | To summarize existing research syntheses reporting newly graduated registered nurses' experiences of providing direct care in hospital settings | | 9 | | Qualitative reviews | | Qualitative studies |
| Carsley 2024 | 1. The primary objective of this work; therefore, was to examine the impact of the COVID-19 pandemic, specifically the public health measures set in place to reduce infection rates, on the five adverse childhood experiences defining child abuse (physical, emotional, sexual) and neglect (physical and emotional) among children under the age of 18 years. 2. The secondary objective was to review author recommendations on how to avoid the unintended impacts of future emergency lockdown procedures on children's health | | 11 | | Quantitative reviews | | Cross sectional studies; Case series/case reports |
| Ell 2023 | To provide a systematic umbrella review that focuses exclusively on alternative treatment options for insomnia disorder by comparing the efficacy of melatonin, light exposure therapy, exercise, and complementary and alternative medicine for insomnia disorder with respect to placebo (e.g., sham acupuncture, sleep hygiene) or waiting-list control conditions in adults | | 15 | | Quantitative reviews; Other: network meta-analyses and meta-analyses | | Randomized controlled trials |
| Seretis 2023 | The aim of this overview was to summarize the available evidence from the existing pool of systematic reviews and meta-analyses, focusing on Vitamin D (VitD) serum status, dietary intake, and VitD receptor (VDR) polymorphisms in correlation to non-melanoma skin cancer incidence. | | 10 | | Quantitative reviews | | Randomized controlled trials |
| Rodolico 2024 | The aim of this study is to consolidate the rapidly growing body of literature on the efficacy and safety of ketamine and esketamine on unipolar and bipolar depression using standard criteria | | 26 | | Quantitative reviews | | Randomized controlled trials |
| Padilla-Cá¡ceres 2023 | To determine the association between the risk of preterm birth and low birth weight in newborns and periodontal disease in pregnant women. | | 15 | | Quantitative reviews | | Cohort studies; case-control; cross sectional studies; quasi-experimental studies |
| Pathomwichaiwat 2023 | Aims to comprehensively review the existing evidence and conduct analysis of updated randomized controlled trials (RCTs) of turmeric (Curcuma longa, CL) and its related bioactive compounds on glycemic and metabolic parameters in patients with type 2 diabetes (T2DM), prediabetes, and metabolic syndrome (MetS) together with a sub-group analysis of different CL preparation forms. | | 14 | | Quantitative reviews | | Randomized controlled trials |
| Talebi 2023 | This umbrella review aims to examine systematic reviews to determine the effectiveness of pro-, pre-, and synbiotics on hormonal parameters, glycemic control markers, blood lipids, anthropometric indices, and inflammatory and oxidative stress biomarkers in women with PCOS and update the evidence. | | 9 | | Quantitative reviews | | Randomized controlled trials |
| AlQahtani 2023 | Aimed to review the current evidence on the efficacy of Tranexamic acid in patients undergoing orthognathic surgery. | | 7 | | Quantitative reviews | | Randomized controlled trials; Cohort studies |
| Brusola 2023 | To conduct a comprehensive umbrella review of systematic reviews evaluating current physical therapy interventions on spasticity outcomes in adults post-stroke. | | 8 | | Quantitative reviews | | Randomised controlled trials; Quasi-experimental studies |
| Liguori 2023 | The effectiveness of rehabilitation interventions for individuals with cerebral palsy | | 8 | | Quantitative reviews | | Randomized controlled trials |
| Ma 2024 | To evaluate the existing evidence and offer a comprehensive understanding of the associations between the use of Red Yeast Rice preparations and various adverse health outcomes | | 15 | | Quantitative reviews | | Randomized controlled trials |
| Li 2024 | To evaluate and summarize systematic reviews of the effects and safety of awake prone positioning for COVID-19-related acute hypoxaemic respiratory failure | | 11 | | Quantitative reviews; Other: meta-analyses | | Randomized controlled trials; Cohort studies |
| Najafi 2023 | Examined the relationship between metformin consumption to treat diabetes and the risk of developing cancer | | 36 | | Quantitative reviews; Other: Meta-analyses | | Randomized controlled trials; Cohort studies; Case control studies |
| Singh 2023 | Aims to consolidate evidence from systematic reviews of the association of common autoimmune conditions with pregnancy outcomes in order to identify the strength and precision of these associations. | | 32 | | Quantitative reviews | | Cohort studies; Case control studies |
| Plutecki 2023 | The main objective of the present umbrella review was to summarize and analyze all of the evidence-based data provided by numerous meta-analyses and systematic reviews regarding the treatment of oropharyngeal squamous cell carcinoma. Our study delivers the most up-to-date and evidence-based results regarding the different therapeutic modalities of this malignancy in one concise review, making it the ultimate tool for physicians treating OPSCC | | 28 | | Quantitative reviews | | Case control studies |
| Ayano 2023 | (1) The global prevalence estimates of Attention-Deficit/Hyperactivity Disorder (ADHD) in children and adolescents; (2) the prevalence estimates specifically in boys and girls; and (3) the prevalence estimates for the three subtypes of ADHD. | | 13 | | Quantitative reviews | | Prevalence studies; Other: not specified however, any study that included a prevalence - quant only. |
| AnandPrakash 2024 | The primary goal of the present study was locating, summarizing, and synthesizing information from published reviews dealing with the epidemiology of musculoskeletal discomfort and injury in dancers around the world. A secondary goal was to make suggestions for upcoming practice and research. | | 12 | | Quantitative reviews | | Cohort studies; Cross sectional studies |
| Xu 2023 | An umbrella review was conducted to investigate the effect of Pilates on multiple health outcomes by systematically collecting and evaluating existing evidence from SRs with MAs | | 27 | | Quantitative reviews | | Randomized controlled trials; Quasi-experimental studies |
| Reis 2023 | What data allow the evaluation of patients with ventilatory compromisation and support nonpharmacological interventions? What is the efficacy of the nonpharmacological interventions in the ventilatory domain, namely, “training of the respiratory muscle”, “execution of techniques of breathing control”, “execution of positioning techniques”, and “execution of thoracic expansion techniques”? | | 44 | | Quantitative reviews | | Randomized controlled trials; Quasi-experimental studies |
| SeyedAlinaghi 2024 | The main purpose of this study is to investigate the effect of macronutrient and micronutrient supplements on COVID-19. | | 28 | | Quantitative reviews | | Randomized controlled trials; Quasi-experimental studies; Cohort studies; Case control studies; Cross sectional studies; Case series/case reports |
| D'Ambrosio 2023 | To evaluate what are the most common medications and systemic diseases that can affect bone implant integration, the success rate and survival rate of dental implants, peri-implant tissue health, and implant loss. | | 8 | | Quantitative reviews | | Randomized controlled trials; Cohort studies; Case control studies; Cross sectional studies; quasi-experimental studies |
| Petrelli 2023 | To assess the strength and quality of evidence and graded the evidence derived from published meta-analyses on this subject. | | 15 | | Quantitative reviews | | Randomized controlled trials; Cohort studies; Case series/case reports |
| Singh 2023 | 1. Aimed to integrate previous systematic review and meta-analytic findings on the earliest neurobiological mechanisms of depression, including dysfunction in the brain, in the Hypothalamic-Pituitary-Adrenal axis and immune/inflammatory systems, in genetics, epigenetics, and other environmental diatheses that are compared in individuals with and without early-onset depression. 2. If the extracted data permit further analysis of moderator terms, we also aimed to determine whether the magnitude of the association between specific diatheses and early-onset depression was affected by methodological (e.g., study design, reporter, time lag between exposure and outcome) and/or observed (e.g., sex or gender, age, socioeconomic status, family history) variables. | | 89 | | Quantitative reviews | | Quasi-experimental studies; Cohort studies; Case control studies; Cross sectional studies; Other: Multiple aggregated and nonredundant studies |
| Huggard 2023 | What are the social determinants of mental illness? | | 37 | | Quantitative reviews; Qualitative reviews; Mixed methods (quant+ qual) reviews | | Randomized controlled trials; Quasi-experimental studies; Cohort studies; Case control studies; Cross sectional studies; Case series/case reports; Qualitative studies; Mixed methods studies; Text and opinion |
| Xue 2024 | To evaluate the effectiveness of repetitive transcranial magnetic stimulation (rTMS) as a treatment for Alzheimer's disease by summarizing the evidence from SRs and MAs | | 8 | | Quantitative reviews | | Not mentioned |
| Snoswell 2023 | The aim of this review is to determine if mortality rates change when telehealth is compared with usual care | | 24 | | Quantitative reviews | | Not mentioned |
| Li 2023 | To evaluate the reporting and methodological quality, and to grade the available evidence of associations between N. sativa and health outcomes | | 20 | | Quantitative reviews | | Randomized controlled trials |
| Huggins 2023 | Examines non-professional interventions that have been implemented to support family caregivers of older adults with dementia who are living in the community. | | 19 | | Quantitative reviews | | Not mentioned |
| Pantiru 2024 | To what extent do gardening and horticultural interventions reduce the risk of poor well-being, mental health, and quality of life? 2. Do the benefits of gardening and horticultural interventions depend on timing, intensity, or duration of activities? | | 40 | | Quantitative reviews | | Randomized controlled trials |
| Lappas 2024 | To evaluate the efficacy and safety of antidepressants in treating sleep disturbances in patients with posttraumatic stress disorder. | | They did not report SRs but reported 39 RCTs included | | Quantitative reviews; Other: They focus on the RCTs, not the SR | | Randomized controlled trials; Case series/case reports |
| Zhao 2023 | To summarize the contents and evaluate the effects of non-pharmacological interventions to prevent and treat delirium among older people. | | 24 | | Quantitative reviews; Other: meta-analyses | | Not mentioned |
| Gu 2024 | To synthesize the available evidence on the effectiveness of single-lead electrocardiogram devices in detecting Atrial Fibrillation. | | 8 | | Quantitative reviews | | Randomized controlled trials; Cohort studies; Case control studies; Cross sectional studies; |
| Dörfler 2023 | Our aim was to critically assess the evidence presented in SRs and meta- MAs about the effectiveness of acupuncture on radiotherapy induced xerostomia. | | 8 | | Quantitative reviews; Other: meta-analyses | | Randomized controlled trials; Quasi-experimental studies; Cohort studies; Case control studies |
| Salem 2024 | To systematically review, summarize, and appraise the findings of published systematic reviews with or without meta-analyses that investigate the effects of branched-chain amino acids on post-exercise recovery of muscle damage biomarkers, muscle soreness, and muscle performance. The secondary objective was to re-analyze and standardize the results of meta-analyses using the random-effects Hartung-Knapp-Sidik-Jonkman method | | 10 | | Quantitative reviews | | Randomized controlled trials |
| Zhang 2023 | To systematically collect and evaluate data on red/processed meat consumption and non-cancer-related outcomes and provide comprehensive evidence | | 40 | | Quantitative reviews | | Cohort studies; case-control studies; cross sectional studies |
| Lewis 2023 | To summarise the evidence from Cochrane Reviews and other systematic reviews of randomised or quasi-randomised trials evaluating the effects of pharmacological and non-pharmacological interventions, administered perioperatively, on reducing blood loss, anaemia, and the need for ABT in adults undergoing hip fracture surgery | | 26 | | Quantitative reviews | | Randomized controlled trials; Quasi-experimental studies |
| Aljuwaiser 2023 | To summarise the prevalence of common mental health problems and interventions for junior doctors and medical students from existing published systematic reviews | | 36 | | Quantitative reviews | | Randomized controlled trials; Quasi-experimental studies; Cohort studies; Mixed methods studies |
| Marques 2023 | What are the clinical outcomes of dental implants in head and neck cancer patients? | | 20 | | Quantitative reviews | | Not mentioned |
| Diegoli 2023 | Summarizes the published SR on the effectiveness and safety of  transcatheter aortic valve replacement (TAVR) compared to surgical aortic valve replacement (SAVR), facilitating decision-making when choosing between TAVR and SAVR or between TAVR devices. | | 60 | | Quantitative reviews | | Randomized controlled trials; Other: Observational studies |
| Chen 2023 | To focus on this topic with multifaceted evaluation and quantitative synthesis. To make clear the consistency, quality, and certainty of evidence, MAs on this topic were collected and assessed comprehensively, and evidence maps with new results were conducted based on different concomitant medications, which may provide a comprehensive and intuitive reference value for clinical application of medications during immunotherapy period in non-small cell lung cancer | | 15 | | Quantitative reviews | | Randomized controlled trials; Cohort studies; Case control studies |
| Eyeberu 2024 | To consolidate many findings of systematic review and meta-analysis studies on neonatal sepsis into a single comprehensive publication where the findings of these reviews could be compared and contrasted | | 5 | | Quantitative reviews; Other: meta-analyses | | Cohort studies; Case control studies; Cross sectional studies |
| Romantsik 2023 | To summarize the evidence from systematic reviews regarding the effects and safety of pharmacological interventions related to pain and sedation management in order to prevent  Germinal matrix hemorrhage and intraventricular hemorrhage in ventilated preterm infants | | 7 | | Quantitative reviews | | Randomized controlled trials |
| Ambagtsheer 2023 | To perform an umbrella review of systematic reviews with MAs examining the effectiveness of comprehensive geriatric assessment delivered within community settings to general populations of community-dwelling older people against various health outcomes. | | 11 | | Quantitative reviews | | Randomized controlled trials |
| Keller 2024 | To systematically evaluate and summarize evidence across multiple SRs examining interventions addressing polypharmacy | | 14 | | Quantitative reviews | | Randomized controlled trials; Quasi-experimental studies; Cohort studies; Cross sectional studies; Other: some mention 'observational studies' but don't specify specific designs exploratory |
| Heredia-Rizo 2023 | To gather evidence on the effectiveness and safety of qigong, tai chi, and yoga to modulate symptoms associated with chronic respiratory diseases | | 27 | | Quantitative reviews | | Randomized controlled trials |
| Liu 2023 | We conducted an umbrella review, which incorporated verification spanning a number of meta-analyses and systematic reviews to clarify the link that existed between PS consuming and health outcomes. | | 23 | | Quantitative reviews | | Randomized controlled trials |
| Rotini 2023 | The primary aim of this study was to assess the short- and long-term functional outcomes and pain scores between arthroscopic surgery and exercise therapy for the treatment of degenerative meniscal lesions in middle-aged patients. The secondary aim was to evaluate the methodological quality and summarize the results of the most recent systematic reviews on surgical or conservative treatments for degenerative meniscal lesions | | 13 | | Quantitative reviews | | Randomized controlled trials; Other: prospective studies |
| Cruciani 2023 | To reappraise the validity of systematic reviews and meta-analyses related to the performance of Aspergillus polymerase chain reaction tests for the diagnosis of invasive aspergillosis inimmunocompromised patients | | 8 | | Quantitative reviews; Other: meta-analyses | | Cohort studies; Case control studies |
| Teshale 2023 | To provide the first comprehensive overview of the current evidence in the role of social determinants of health in Cardiovascular Diseases by undertaking an umbrella review. | | 70 | | Quantitative reviews | | Randomized controlled trials; Quasi-experimental studies; Cohort studies; Case control studies; Cross sectional studies; Case series/case reports |
| AmiriKhosroshahi 2023 | To examine the effect of nutritional interventions on varying severity of cancer therapy-induced oral mucositis and rank the certainty of obtained evidence | | 26 | | Quantitative reviews | | Randomized controlled trials |
| Lin 2024 | To provide a comprehensive evaluation of the evidence on lifestyle medicine and integrative therapies for inflammatory arthritis. | | 52 | | Quantitative reviews | | Randomized controlled trials |
| Li 2023 | The existing SRs/MA) were re-evaluated to provide a reference for the clinical application of Compind Kushen injection | | 18 | | Quantitative reviews | | Randomized controlled trials |
| Xu 2023 | A comprehensive review of environmental risk, protective factors, and biomarkers for AR to establish the evidence hierarchy. | | 42 | | Quantitative reviews | | Cohort studies; cross-sectional studies; case–control studies |
| Li 2023 | Our aim was to synthesize both qualitative and quantitative evidence on prevalence and outcomes of long-term effect of COVID-19 through an umbrella review | | 36 | | Quantitative reviews | | Cohort studies; Case control studies; Cross sectional studies; Case series/case reports; Prevalence studies |
| Palella 2023 | This umbrella review aimed to summarize the epidemiologic evidence and evaluate its strength and validity on the associations of Graves' disease with thyroid cancer risk and its prognosis. | | 5 | | Quantitative reviews | | Cohort studies; Case control studies |
| Smit 2023 | To provide a comprehensive and systematic overview of the literature to assess the importance and evidence related to variables for resistance and the temporal relationship between variables and resistance development for the community through an umbrella review. To provide an overall picture of the findings for a particular phenomenon. | | 23 | | Quantitative reviews | | Randomized controlled trials; Cohort studies; Case control studies |
| Laplaud 2023 | To summarize the effectiveness of yoga interventions on PTSD symptoms in adults in a SR including randomized controlled trials (RCTs). | | 11 | | Quantitative reviews | | Randomized controlled trials |
| Benyamini 2024 | The aim of the current review of reviews was to identify key dimensions of women and partners childbirth experiences in order to improve the understanding and assessment of childbirth experiences and guide interventions aimed at promoting a positive birth experience | | 40 | | Qualitative reviews; | | Not mentioned |
| Solmi 2023 | To systematically assess credibility and certainty of associations between cannabis, cannabinoids, and cannabis based medicines and human health, from observational studies and randomised controlled trials | | 101 | | Quantitative reviews; Other: meta-analyses | | Randomized controlled trials; Quasi-experimental studies; Cohort studies; Case control studies |
| Zhang 2023 | We aimed to systematically review relationships between gut microbiome dysbiosis and diseases and determine evidence on fecal microbiota transplantation efficacy and safety for various indications. | | 16 | | Quantitative reviews | | Randomized controlled trials; Cohort studies |
| McEvoy 2023 | Examined the risk and protective factors for self-harm in young people. | | 9 | | Quantitative reviews; Mixed methods (quant+ qual) reviews | | Qualitative studies; (only mentions quantitative studies not designs) |
| Law 2023 | To review the mental health outcomes of bariatric surgery to reveal the quality and strength of the evidence. | | 9 | | Quantitative reviews | | Randomised controlled trials; cohort; case control |
| McCready 2023 | To explore the barriers and facilitators of vaccine hesitancy toward the COVID-19 vaccine for healthcare workers and healthcare students. | | 31 | | Quantitative reviews | | Not mentioned |
| Yaow 2023 | With the growing evidence from systematic reviews examining the sex differences in cardiovascular outcomes for patients with Type 2 Diabetes Mellitus, an umbrella review is an essential next step to synthesising and assessing the strength of the available evidence. | | 27 | | Quantitative reviews | | Not mentioned |
| Aljuraiban 2023 | To evaluate the association between these dietary factors and risk of hypertension with change in blood pressure | | 175 publications reporting 341 meta-analyses o | | Quantitative reviews | | Randomized controlled trials; Cohort studies |
| Niño-de-GuzmanQuispe 2023 | Aimed to identify the most cost-effective self management interventions for type 2 diabetes mellitus, among other chronic disease | | 54 | | Quantitative reviews; Qualitative reviews; Mixed methods (quant+ qual) reviews | | Other: They mentioned only the review as their primary studies as indicated above. |
| Perez-Dominguez 2023 | To review the effects of resistance training on patients with end-stage renal disease and assess the methodological quality of the available literature | | 24 | | Quantitative reviews | | Randomized controlled trials; Quasi-experimental studies |
| VieiraNascimento 2023 | To systematically summarize the evidence for photobiomodulation therapy (PBMT) in the prevention and treatment of oral mucositis (OM) in patients undergoing cancer treatment. | | 16 | | Quantitative reviews | | Randomized controlled trials; Quasi-experimental studies |
| Fazel 2024 | To provide a comprehensive overview of the current evidence regarding the effectiveness of different psychosocial interventions in reducing all forms of violence toward others. | | 116 papers (30 meta-analyses) | | Quantitative reviews | | Randomized controlled trials |
| Jain 2024 | To provide more clarity on the type of interventions that are effective in improving routine immunisation outcomes for children in low and middle income countries. | | 62 | | Quantitative reviews; Qualitative reviews; Mixed methods (quant+ qual) reviews | | Not mentioned |
| Emile 2023 | The present study aimed to provide an overview of the collective evidence on the association between coffee consumption and risk of colorectal cancer | | 14 | | Quantitative reviews | | Cohort studies; Case control studies |
| Kar 2023 | Examine the safety and efficacy of repetitive transcranial magnetic stimulation in Obsessive compulsive disorder treatment | | 12 | | Quantitative reviews; Other: Meta-analyses | | Randomized controlled trials |
| McDermott 2023 | Aimed to synthesise the evidence for the effectiveness of digital interventions on consumption of unhealthy food/Sugar Sweetened Beverages (SSBs)and physical inactivity in adolescents and young people. Our objective was to ascertain (1) are digital interventions aimed at young people effective in addressing physical inactivity and poor dietary choices? and (2) What is the quality and strength of the systematic review evidence? | | 49 | | Quantitative reviews | | Randomized controlled trials; Quasi-experimental studies; Not mentioned |
| Rasaei 2024 | Aims to give a snapshot of the influence of prebiotic, probiotic, or synbiotic intake on body weight changes, irrespective of age and sex differences | | 97 | | Quantitative reviews; Other: or meta-analyses | | Randomized controlled trials |
| Wnuk 2023 | The purpose of this study is to identify and evaluate the effectiveness of workplace interventions aimed at preventing type 2 diabetes that can implemented in the workplace. | | 7 | | Quantitative reviews | | Randomized controlled trials; Other: pre, post, pre-post studies |
| Patikorn 2023 | To systematically identify relevant meta-analyses of RCTs of ketogenic diets (KD), summarize their findings, and assess the strength of evidence of the effects of KD on health outcomes | | 17 | | Quantitative reviews; Other: meta-analyses | | Randomized controlled trials |
| Sufrate-Sorzano et al 2023 | To determine which interventions, from a nursing perspective, can be considered as the interventions of choice for the prevention and treatment of suicidal behaviour. | | 21 | | Quantitative reviews | | Randomized controlled trials |
| Phillips 2024 | To identify and appraise higher level evidence (systematic reviews with or without meta-analyses) reporting on the association between migraine and pregnancy outcomes, to consolidate evidence from systematic reviews and meta-analyses using narrative synthesis and, where appropriate, quantitative synthesis, and to update existing systematic reviews and meta-analyses | | 4 | | Quantitative reviews | | Randomized controlled trials; Cohort studies; Case control studies; Cross sectional studies |
| Babot-Pereña 2023 | To explain the current evidence and demonstrate the most effective management of split-thickness skin grafts donor sites. The specific objectives are to evaluate the efficacy of different donor site healing techniques in improving re-epithelialization, pain, and incidence of infection, and to make recommendations | | 5 | | Quantitative reviews | | Randomized controlled trials; Case control studies; Other: 1 randomised uncontrolled |
| Eost-Telling 2024 | The aim of this overview is to evaluate evidence for any types of technologies that aim to reduce falls and fall risk for People living with dementia or mild cognitive impairment and to identify gaps in the evidence base. | | 7 | | Quantitative reviews | | Other: not stated |
| Sun 2024 | To summarize evidence from systematic reviews SRs/MAs regarding the impact of dyadic interventions delivered to both members of a cancer dyad, including a cancer patient and caregiver (e.g. family caregiver, intimate partner) | | 18 | | Quantitative reviews; Other: meta-analyses | | Randomized controlled trials; Quasi-experimental studies |
| Wnuk 2023 | The aim of this study is to summarise the effectiveness of fissure sealing of permanent teeth with fissure sealants compared to other preventive methods or no intervention. | | 15 | | Quantitative reviews | | Randomized controlled trials |
| SalazardePablo 2024 | To summarize, for the first time, the meta-analytical evidence in the field of this vulnerable population and to provide evidence-based recommendations. | | 30 | | Quantitative reviews | | Not mentioned |
| Zhou 2023 | (1) Identify barriers and facilitators for clinical practice guideline implementation; (2) map them to the Theoretical domains framework: and (3) map them to the Behavior Change Wheel | | 37 | | Quantitative reviews; Qualitative reviews; Mixed methods (quant+ qual) reviews | | Randomized controlled trials; Quasi-experimental studies; Cohort studies; Cross sectional studies; Case series/case reports; Qualitative studies; Mixed methods studies; Economic evaluation studies; Other |
| Sufrate-Sorzano 2023 | What nursing interventions have been shown to be effective in the prevention and treatment of suicidal behavior in adults? | | 21 | | Quantitative reviews | | Not mentioned |

Supplementary 2: Summary of results

| **UR Characteristic** | **Frequency (n) of URs** |
| --- | --- |
| Protocol/registration information available | Yes 66  No 34 |
| Question Framework/Mnemonic used | PICO 43  PICOS 14  PICOTS 1  PICo 4  SPIDER 1  PEO 6  PCC 2  PECOS 1  PECO 1  Population, outcomes and comparator 1  Population, Phenomenon of interest 1  Not specified 25 |
| Methodological guidance followed | Cochrane methodological guidance 28  Cochrane methodological guidance; JBI methodological guidance 1  Cochrane and other 3  JBI 13  Other 3  Not mentioned 55 |
| Reporting guidance followed | PRISMA 73  PRIOR 15  ENTREQ 1  Not mentioned 16  Prio-harms 3  PRISMA-DTA 1  BRC (brief review checklist) 1  PRISMA-ScR 1  Pollock et al., 2017 1 |
| Number of SRs were included in the data synthesis stage | Range 4-175 |
| Types of SRs included | Quantitative 98  Qualitative 2  Mixed methods 7  Meta-analyses 14  Network meta-analysis 1 |
| Types of primary studies were included | RCTs 68  Cohort 36  Case-control 26  Quasi-experimental 24  Cross sectional 20  Prevalence 3  Qualitative 6  Mixed methods 4  Case reports/case series 12  Not mentioned 12 |
| Methods used for the overlap of the primary studies within SRs | Narrative description 16  Corrected covered area 21  Tabular presentation 15  Other 9  Not mentioned 52 |
| Overlap reported for individual outcomes | Yes 13  No 31 |
| Critical appraisal undertaken | Yes 97  No 3 |
| Critical appraisal tools used | AMSTAR-2 69  JBI 10  AMSTAR8  ROBIS10  Other - 10  Not applicable 3 |
| UR that excluded any reviews based on methodological quality | Yes 4 |
| Quantitative data analysis approaches | Narratively 98  Basic Descriptive Statistics 27  Meta-analysis 30  Other - 3  Not applicable 2 |
| Quantitative data presentation approaches | Narrative text only 8  JBI Stop light figure 3  Summary of Findings table 18  Tabular Display 76  Forest Plot/s 29  Other 13 |
| Qualitative data analysis approaches | Narratively 7  Basic descriptive statistics 1  Qualitative synthesis 3  Other 1 |
| Qualitative data presentation approaches | Summary of findings table 2  Tabular display 5  Other 2 |
| Number of figures/tables/plots used in UR | Range 0-32 |
| Certainty of evidence assessed | GRADE 39  CERqual 4  ConQual 1  NutriGRADE 2  Other 7  Certainty not assessed 52 |
| Software of analysis/presentation | Yes 27  No 73 |
| Additional data presented in supplementary files | Yes 51  No 49 |

Supplementary 3: Citation details of included reviews

| **Title** | **Authors** | **Published Year** | **Journal** | **Volume** | **Issue** | **Pages** |
| --- | --- | --- | --- | --- | --- | --- |
| Umbrella review of nursing interventions NIC for the treatment and prevention of suicidal behavior. | Sufrate-Sorzano, Teresa; Perez, Jesus; Juarez-Vela, Raul; Garrote-Camara, MariaElena; de Vinaspre, Regina Ruiz; Molina-Luque, Fidel; Santolalla-Arnedo, Ivan | 2023 | International journal of nursing knowledge | 34 | 3 | 204-215 |
| The barriers and facilitators for the implementation of clinical practice guidelines in healthcare: an umbrella review of qualitative and quantitative literature. | Zhou, Pengxiang; Chen, Lu; Wu, Ziyang; Wang, Ente; Yan, Yingying; Guan, Xiaodong; Zhai, Suodi; Yang, Kehu | 2023 | Journal of clinical epidemiology | 162 |  | 169-181 |
| Umbrella Review: Atlas of the Meta-Analytical Evidence of Early-Onset Psychosis | Salazar de Pablo, G; Rodriguez, V; Besana, F; Civardi, SC; Arienti, V; Marañ a Garceo, L; Andrés-Camazón, P; Catalan, A; Rogdaki, M; Abbott, C | 2024 | Journal of the American Academy of Child and Adolescent Psychiatry | 63 | 7 | 684-697 |
| Evaluation of the effectiveness of prophylactic sealing of pits and fissures of permanent teeth with fissure sealants-umbrella review | Wnuk, Katarzyna; Åšwitalski, Jakub; Miazga, Wojciech; Tatara, Tomasz; Religioni, Urszula; Gujski, Mariusz | 2023 | BMC Oral Health | 23 | 1 | 806 |
| Effectiveness of dyadic interventions among cancer dyads: An overview of systematic reviews and meta-analyses | Sun, Qian; Wang, Kunyuan; Chen, Yingliang; Peng, Xiuhua; Jiang, Xiaohan; Peng, Junsheng | 2024 | Journal of Clinical Nursing | 33 | 2 | 497-530 |
| Digital technologies to prevent falls in people living with dementia or mild cognitive impairment: a rapid systematic overview of systematic reviews | Eost-Telling, Charlotte; Yang, Yang; Norman, Gill; Hall, Alex; Hanratty, Barbara; Knapp, Martin; Robinson, Louise; Todd, Chris | 2024 | Age and ageing | 53 | 1 |  |
| Healing techniques for split-thickness skin grafts donor sites. Umbrella review | Babot-Pereña Núria; Blanco-Blanco, Joan | 2023 | Enferm Clin (Engl Ed) | 33 | 6 | 432-437 |
| How migraine and its associated treatment impact on pregnancy outcomes: Umbrella review with updated systematic review and meta-analysis | Phillips, Katherine; Clerkin-Oliver, Conor; Nirantharakumar, Krishnarajah; Crowe, Francesca L; Wakerley, Benjamin R | 2024 | Cephalalgia | 44 | 2 |  |
| Interventions of choice for the prevention and treatment of suicidal behaviours: An umbrella review | Sufrate-Sorzano, Teresa; Santolalla-Arnedo, Ivan; Garrote-Cámara, María Elena; Angulo-Nalda, Beatriz; Cotelo-Sáenz, Ruth; Pastells-Peiró , Roland; Bellon, Filip; Blanco-Blanco, Joan; Juárez-Vela, Molina-Luque, Fidel | 2023 | Nursing open | 10 | 8 | 4959-4970 |
| Effects of ketogenic diet on health outcomes: an umbrella review of meta-analyses of randomized clinical trials | Patikorn, Chanthawat; Saidoung, Pantakarn; Pham, Tuan; Phisalprapa, Pochamana; Lee, Yeong Yeh; Varady, Krista A; Veettil, Sajesh K; Chaiyakunapruk, Nathorn | 2023 | BMC medicine | 21 | 1 | 196 |
| Workplace Interventions for Type 2 Diabetes Mellitus Prevention - an Umbrella Review | Wnuk, Katarzyna; Åšwitalski, Jakub; Tatara, Tomasz; Miazga, Wojciech; Jopek, Sylwia; Augustynowicz, Anna; Religioni, Urszula; Gujski, Mariusz | 2023 | Current Diabetes Reports | 23 | 10 | 293-304 |
| The effects of prebiotic, probiotic or synbiotic supplementation on overweight/obesity indicators: an umbrella review of the trials’ meta-analyses | Rasaei, Niloufar; Heidari, Mohammadreza; Esmaeili, Fataneh; Khosravi, Sepehr; Baeeri, Maryam; Tabatabaei-Malazy, Ozra; Emamgholipour, Solaleh | 2024 | Frontiers in Endocrinology | 15 |  | 1E+06 |
| Digital interventions to moderate physical inactivity and/or nutrition in young people: a Cancer Prevention Europe overview of systematic reviews | McDermott, Kevin T; Noake, Caro; Wolff, Robert; Bauld, Linda; Espina, Carolina; Foucaud, Jérôme; Steindorf, Karen; Thorat, Mangesh A; Weijenberg, Matty P; Schüz, Joachim | 2023 | Frontiers in digital health | 5 |  | 1E+06 |
| The efficacy of transcranial magnetic stimulation in the treatment of obsessive-compulsive disorder: an umbrella review of meta-analyses | Kar, Sujita Kumar; Agrawal, Aditya; Silva-dos-Santos, Amílcar; Gupta, Yogesh; Deng, Zhi-De | 2023 | CNS spectrums | 29 | 2 |  |
| Does drinking coffee reduce the risk of colorectal cancer? A qualitative umbrella review of systematic reviews | Emile, SH; Barsom, SH; Garoufalia, Z; Wexner, SD | 2023 | Techniques in Coloproctology | 27 | 11 | 961-968 |
| Effective interventions for improving routine childhood immunisation in low and middle-income countries: a systematic review of systematic reviews | Jain, Monica; Duvendack, Maren; Shisler, Shannon; Parsekar, Shradha S; Leon, Maria Daniela Anda | 2024 | BMJ open | 14 | 2 | e074370 |
| Effectiveness of violence prevention interventions: Umbrella review of research in the general population | Fazel, Seena; Burghart, Matthias; Wolf, Achim; Whiting, Daniel; Yu, Rongqin | 2024 | Trauma, Violence, & Abuse | 25 | 2 | 1709-1718 |
| Management of cancer therapy-induced oral mucositis using Photobiomodulation therapy: an overview of systematic reviews | Vieira Nascimento, Matheus; Costa, Fabio Wildson Gurgel; de Oliveira Filho, Osias Vieira; Silva, Paulo GoberlÃ¢nio de Barros; de Freitas Pontes, Karina Matthes | 2023 | Photobiomodulation, Photomedicine, and Laser Surgery | 41 | 10 | 513-538 |
| Effects of resistance training on patients with End-Stage Renal Disease: an umbrella review with meta-analysis of the pooled findings | Perez-Dominguez, Borja; Suso-Marti, Luis; Dominguez-Navarro, Fernando; PerpiÃ±a-Martinez, Sara; Calatayud, Joaquin; CasaÃ±a, Jose | 2023 | Journal of Nephrology | 36 | 7 | 1805-1839 |
| Patients’ and informal caregivers’ perspectives on self-management interventions for type 2 diabetes mellitus outcomes: a mixed-methods overview of 14 years of reviews | NiÃ±o-de-Guzman Quispe, Ena; Bracchiglione, Javier; Ballester, Marta; Groene, Oliver; Heijmans, Monique; MartÃ­nez GarcÃ­a, Laura; Noordman, Janneke; Orrego, Carola; Rocha, Claudio; SuÃ±ol, Rosa | 2023 | Archives of Public Health | 81 | 1 | 140 |
| The role of diet in the prevention of hypertension and management of blood pressure: An umbrella review of meta-analyses of interventional and observational studies | Aljuraiban, Ghadeer S; Gibson, Rachel; Chan, Doris SM; Van Horn, Linda; Chan, Queenie | 2023 | Advances in Nutrition | 15 | 1 |  |
| Higher risk of adverse cardiovascular outcomes in females with type 2 diabetes Mellitus: an Umbrella review of systematic reviews | Yaow, Clyve Yu Leon; Chong, Bryan; Chin, Yip Han; Kueh, Martin Tze Wah; Ng, Cheng Han; Chan, Kai En; Tang, Ansel Shao Pin; Chung, Charlotte; Goh, Rachel; Kong, Gwyneth | 2023 | European Journal of Preventive Cardiology | 30 | 12 | 1227-1235 |
| Understanding the barriers and facilitators of vaccine hesitancy towards the COVID-19 vaccine in healthcare workers and healthcare students worldwide: An Umbrella Review | McCready, Jemma Louise; Nichol, Bethany; Steen, Mary; Unsworth, John; Comparcini, Dania; Tomietto, Marco | 2023 | PLoS One | 18 | 4 | e0280439 |
| The effectiveness, implementation, and experiences of peer support approaches for mental health: a systematic umbrella review | Cooper, Ruth E; Saunders, Katherine RK; Greenburgh, Anna; Shah, Prisha; Appleton, Rebecca; Machin, Karen; Jeynes, Tamar; Barnett, Phoebe; Allan, Sophie M; Griffiths, Jessica | 2024 | BMC medicine | 22 | 1 | 72 |
| Risk and protective factors for self-harm in adolescents and young adults: an umbrella review of systematic reviews | McEvoy, David; Brannigan, Ross; Cooke, Lorcan; Butler, Emma; Walsh, Cathal; Arensman, Ella; Clarke, Mary | 2023 | Journal of psychiatric research | 168 |  | 353-380 |
| The gut microbiome dysbiosis and regulation by fecal microbiota transplantation: umbrella review | Zhang, Xianzhuo; Luo, Xufei; Tian, Liang; Yue, Ping; Li, Mengyao; Liu, Kefeng; Zhu, Daoming; Huang, Chongfei; Shi, Qianling; Yang, Liping | 2023 | Frontiers in Microbiology | 14 |  |  |
| Balancing risks and benefits of cannabis use: umbrella review of meta-analyses of randomised controlled trials and observational studies | Solmi, Marco; De Toffol, Marco; Kim, Jong Yeob; Choi, Min Je; Stubbs, Brendon; Thompson, Trevor; Firth, Joseph; Miola, Alessandro; Croatto, Giovanni; Baggio, Francesca | 2023 | bmj | 382 |  |  |
| Key dimensions of women's and their partners’ experiences of childbirth: A systematic review of reviews of qualitative studies | Benyamini, Yael; Delicate, Amy; Ayers, Susan; Dikmen-Yildiz, Pelin; Gouni, Olga; Jonsdottir, Sigridur Sia; Karlsdottir, Sigfridur Inga; Kömürcü Akik, Burcu; Leinweber, Julia; Murphy-Tighe, Sylvia | 2024 | Plos one | 19 | 3 | e0299151 |
| Management of post-traumatic stress disorder symptoms by yoga: an overview | Laplaud, Nina; Perrochon, Anaïck; Gallou-Guyot, Matthieu; Moens, Maarten; Goudman, Lisa; David, Romain; Rigoard, Philippe; Billot, Maxime | 2023 | BMC complementary medicine and therapies | 23 | 1 | 258 |
| One Health Determinants of Escherichia coli Antimicrobial Resistance in Humans in the Community: An Umbrella Review | Smit, Chloé CH; Lambert, Maarten; Rogers, Kris; Djordjevic, Steven P; Van Oijen, Antoine M; Keighley, Caitlin; Taxis, Katja; Robertson, Hamish; Pont, Lisa G | 2023 | International Journal of Molecular Sciences | 24 | 24 | 17204 |
| Risk and prognosis of thyroid cancer in patients with Graves’ disease: an umbrella review | Palella, Marco; Giustolisi, Francesca Maria; Modica Fiascaro, Adriana; Fichera, Martina; Palmieri, Antonella; Cannarella, Rossella; Calogero, Aldo E; Ferrante, Margherita; Fiore, Maria | 2023 | Cancers | 15 | 10 | 2724 |
| Long-COVID-19 clinical and health outcomes: an umbrella review | Li, Hu; Xia, Jin; Bennett, Dimitri; Roque, Fatima; Bam, Rujuta A; Tavares, Ana BÃ¡rbara Tadeu; Gokhale, Mugdha; Ida, Fidelia; Rhee, Jinnie Jiwon; Soriano Gabarro, Montse | 2023 | Therapeutic Advances in Infectious Disease | 10 |  |  |
| Environmental risk factors, protective factors, and biomarkers for allergic rhinitis: a systematic umbrella review of the evidence | Xu, Xianpeng; Liu, Xinghong; Li, Jiongke; Deng, Xinxing; Dai, Tianrong; Ji, Qingjie; Xiong, Dajing; Xie, Hui | 2023 | Clinical Reviews in Allergy & Immunology | 65 | 2 | 188-205 |
| Adjunctive effect of compound Kushen injection for cancer: an overview of systematic reviews | Li, Chenchen; Niu, Dong; Zhu, Ranpei; Yan, Xiaoyu; Qu, Haoran; Zhang, Yaling; Zheng, Yuling | 2023 | Journal of Ethnopharmacology | 317 |  |  |
| The effectiveness and safety of lifestyle medicine and integrative therapies in inflammatory arthritis: an umbrella review using a hierarchical evidence gathering approach | Lin, Joshua; Liu, Jing; Oâ€™Fee, Allana; Pandey, Chhiti; Benna-Doyle, Sarah; Maunder, Alison; Rao, Vibhuti; Alesi, Simon; Ng, Beverly; Ee, Carolyn | 2024 | Frontiers in Medicine | 11 |  |  |
| Nutritional interventions for the prevention and treatment of cancer therapy-induced oral mucositis: an umbrella review of systematic reviews and meta-analysis | Amiri Khosroshahi, Reza; Talebi, Sepide; Zeraattalab-Motlagh, Sheida; Imani, Hossein; Rashidi, Amirabbas; Travica, Nikolaj; Mohammadi, Hamed | 2023 | Nutrition Reviews | 81 | 9 | 1200-1212 |
| The role of social determinants of health in cardiovascular diseases: an umbrella review | Teshale, Achamyeleh Birhanu; Htun, Htet Lin; Owen, Alice; Gasevic, Danijela; Phyo, Aung Zaw Zaw; Fancourt, Daisy; Ryan, Joanne; Steptoe, Andrew; Freak-Poli, Rosanne | 2023 | Journal of the American Heart Association | 12 | 13 | e029765 |
| An overview of systematic reviews of polymerase chain reaction (PCR) for the diagnosis of invasive aspergillosis in immunocompromised people: a report of the fungal PCR initiative (FPCRI)â€”an Isham Working Group | Cruciani, Mario; White, P Lewis; Barnes, Rosemary A; Loeffler, Juergen; Donnelly, J Peter; Rogers, Thomas R; Heinz, Werner J; Warris, Adilia; Morton, Charles Oliver; Lengerova, Martina | 2023 | Journal of Fungi | 9 | 10 | 967 |
| Arthroscopic surgery or exercise therapy for degenerative meniscal lesions: a systematic review of systematic reviews | Rotini, M; Papalia, G; Setaro, N; Luciani, P; Marinelli, M; Specchia, N; Gigante, A | 2023 | Musculoskeletal surgery | 107 | 2 | 127-141 |
| Health outcomes associated with phytosterols: An umbrella review of systematic reviews and meta-analyses of randomized controlled trials | Liu, Bing; Chen, Ke; Chen, Xi; Wang, Jian; Shu, Guangjie; Ping, Zhiguang; Zhang, Shenshen | 2023 | Phytomedicine | 122 |  |  |
| Effectiveness of mind-body exercises in chronic respiratory diseases: an overview of systematic reviews with meta-analyses | Heredia-Rizo, Alberto Marcos; Martinez-Calderon, Javier; Piña-Pozo, Fernando; González-García, Paula; García-Muñoz, Cristina | 2023 | Disability and Rehabilitation | 46 | 12 |  |
| Cumulative update of a systematic overview evaluating interventions addressing Polypharmacy | Keller, Michelle S; Qureshi, Nabeel; Mays, Allison M; Sarkisian, Catherine A; Pevnick, Joshua M | 2024 | JAMA Network Open | 7 | 1 | e2350963-e2350963 |
| Does CGA improve health outcomes in the community? An umbrella review | Ambagtsheer, Rachel C; Thompson, Mark Q; Tucker, Graeme R; Schultz, Timothy; Beilby, Justin; Visvanathan, Renuka | 2023 | Journal of the American Medical Directors Association | 24 | 6 | 782-789 |
| Pharmacological pain and sedation interventions for the prevention of intraventricular hemorrhage in preterm infants on assisted ventilation - an overview of systematic reviews | Romantsik, Olga; Calevo, Maria Grazia; Banzi, Rita; Ley, David; Bruschettini, Matteo | 2023 | Cochrane Database of Systematic Reviews | 8 | 8 |  |
| Neonatal sepsis and its predictors in Ethiopia: umbrella reviews of a systematic review and meta-analysis, 2023 | Eyeberu, Addis; Musa, Ibsa; Debella, Adera | 2024 | Annals of Medicine and Surgery | 86 | 2 | 994-1002 |
| The negative effect of concomitant medications on immunotherapy in non-small cell lung cancer: An umbrella review | Chen, Jixin; Chen, Shuqi; Luo, Huiyan; Long, Shunqin; Yang, Xiaobing; He, Wenfeng; Wu, Wanyin; Wang, Sumei | 2023 | International Immunopharmacology | 124 |  |  |
| Transcatheter Valve Replacement in Patients with Aortic Valve Stenosis: An Overview of Systematic Reviews and Meta-Analysis with Different Populations | Diegoli, Henrique; Alves, Marcia Regina Dias; Okumura, Lucas Miyake; Kroll, Caroline; Silveira, Dayane; Furlan, Luiz Henrique Picolo | 2023 | Arquivos Brasileiros de Cardiologia | 120 |  | e20220701 |
| Clinical outcomes of dental implants in head and neck cancer patients: An overview | Marques, Nelson Pereira; Pérez-de-Oliveira, Maria Eduarda; Normando, Ana Gabriela Costa; Marques, Nádia Carolina Teixeira; Epstein, Joel B; Migliorati, Cesar A; Martelli-Júnior, Hercílio; Ribeiro, Ana Carolina Prado; Rocha, Andre Caroli; Brandão, Thaís Bianca | 2023 | Oral surgery, oral medicine, oral pathology and oral radiology | 136 | 1 | 42-53 |
| Common mental health problems in medical students and junior doctors - an overview of systematic reviews | Aljuwaiser, Sameera; Brazzelli, Miriam; Arain, Imran; Poobalan, Amudha | 2023 | Journal of Mental Health | 33 | 6 | 779-815 |
| Interventions for reducing red blood cell transfusion in adults undergoing hip fracture surgery: an overview of systematic reviews | Lewis, Sharon R; Pritchard, Michael W; Estcourt, Lise J; Stanworth, Simon J; Griffin, Xavier L | 2023 | Cochrane Database of Systematic Reviews | 8 | 6 |  |
| Red/processed meat consumption and non-cancer-related outcomes in humans: Umbrella review | Zhang, Xingxia; Liang, Shiqi; Chen, Xinrong; Yang, Jie; Zhou, Yong; Du, Liang; Li, Ka | 2023 | British Journal of Nutrition | 130 | 3 | 484-494 |
| Branched-chain amino acids supplementation and post-exercise recovery: an overview of systematic reviews | Salem, Atef; Trabelsi, Khaled; Jahrami, Haitham; Alrasheed, Maha M; Boukhris, Omar; Puce, Luca; Bragazzi, Nicola Luigi; Ammar, Achraf; Glenn, Jordan M; Chtourou, Hamdi | 2024 | Journal of the American Nutrition Association | 43 | 4 | 384-396 |
| Umbrella review: Summary of findings for acupuncture as treatment for radiation-induced xerostomia | Dörfler, Jennifer; Freuding, Maren; Zaiser, Christopher; Büntzel, Jens; Keinki, Christian; Käsmann, Lukas; Hübner, Jutta | 2023 | Head & Neck | 45 | 4 | 1026-1044 |
| Effectiveness of single-lead ECG devices for detecting atrial fibrillation: An overview of systematic reviews | Gu, Hai Yue; Huang, Jun; Liu, Xu; Qiao, Shu Qian; Cao, Xi | 2024 | Worldviews on Evidenceâ€Based Nursing | 21 | 1 | 79-86 |
| Non-pharmacological interventions to prevent and treat delirium in older people: An overview of systematic reviews | Zhao, Qin; Liu, Shan; Zhao, Hongyu; Dong, Lei; Zhu, Xiao; Liu, Jia | 2023 | International Journal of Nursing Studies | 148 |  |  |
| Effects of Antidepressants on Sleep in Post-traumatic Stress Disorder: An Overview of Reviews | Lappas, Andreas S; Polyzopoulou, Zoi A; Christodoulou, Nikos; Bozikas, Vasilios-Panteleimon; Samara, Myrto T | 2024 | Current Neuropharmacology | 22 | 4 | 749-805 |
| The impact of gardening on well-being, mental health, and quality of life: an umbrella review and meta-analysis | PanÈ›iru, I; Ronaldson, A; Sima, N; Dregan, A; Sima, R | 2024 | Systematic Reviews | 13 | 1 | 45 |
| Interventions for caregivers of older adults with dementia living in the community: A rapid review of reviews | Huggins, Madison; Pesut, Barbara; Puurveen, Gloria | 2023 | Canadian Journal on Aging/La Revue canadienne du vieillissement | 42 | 3 | 425-433 |
| Nigella sativa and health outcomes: An overview of systematic reviews and meta-analyses | Li, Zhongyu; Wang, Yang; Xu, Qing; Ma, Jinxin; Li, Xuan; Yan, Jiaxing; Tian, Yibing; Wen, Yandong; Chen, Ting | 2023 | Frontiers in Nutrition | 10 |  |  |
| An overview of the effect of telehealth on mortality: A systematic review of meta-analyses | Snoswell, Centaine L; Stringer, Hannah; Taylor, Monica L; Caffery, Liam J; Smith, Anthony C | 2023 | Journal of telemedicine and telecare | 29 | 9 | 659-668 |
| Repetitive transcranial magnetic stimulation for Alzheimer’s disease: an overview of systematic reviews and meta-analysis | Xue, Hua; Li, Ya-xin; Xiao, Ya-song; Fan, Wen-hui; He, Hong-xian | 2024 | Frontiers in Aging Neuroscience | 16 |  |  |
| The social determinants of mental illness: A rapid review of systematic reviews | Huggard, Leigh; Murphy, Rachel; O’Connor, Cliodhna; Nearchou, Finiki | 2023 | Issues in mental health nursing | 44 | 4 | 302-312 |
| Genetics, epigenetics, and neurobiology of childhood-onset depression: an umbrella review | Singh, Manpreet K; Gorelik, Aaron J; Stave, Christopher; Gotlib, Ian H | 2023 | Molecular Psychiatry | 29 | 3 | 553-565 |
| Human papillomavirus infection and non-oropharyngeal head and neck cancers: an umbrella review of meta-analysis | Petrelli, Fausto; Cin, Elisa Dal; Ghidini, Antonio; Carioli, Daniela; Falasca, Vincenzo; De Stefani, Agostina; Moleri, Giovanna; Ardito, Raffaele; Luciani, Andrea; Nardone, Massimiliano | 2023 | European Archives of Oto-Rhino-Laryngology | 280 | 9 | 3921-3930 |
| Do systemic diseases and medications influence dental implant osseointegration and dental implant health? An umbrella review | D’Ambrosio, Francesco; Amato, Alessandra; Chiacchio, Andrea; Sisalli, Laura; Giordano, Francesco | 2023 | Dentistry Journal | 11 | 6 | 146 |
| The effect of macronutrient and micronutrient supplements on COVID-19: an umbrella review | SeyedAlinaghi, SeyedAhmad; Shahidi, Ramin; Mojdeganlou, Hengameh; Akhtaran, Fatemeh Khajeh; Maroufi, Seyed Farzad; Maroufi, Seyede Parmis; Mirzapour, Pegah; Karimi, Amirali; Khodaei, Sepideh; Pour, Mehrzad Mohsseni | 2024 | Journal of Health, Population and Nutrition | 43 | 1 | 16 |
| Effectiveness of Nonpharmacological Interventions in the Field of Ventilation: An Umbrella Review | Reis, Neuza; Gaspar, Luis; Paiva, Abel; Sousa, Paula; Machado, NatÃ¡lia | 2023 | International Journal of Environmental Research and Public Health | 20 | 7 | 5239 |
| Pilates and multiple health outcomes: An umbrella review | Xu, Meng; Tian, Chen; Wang, Yong; Liang, Shanshan; Wang, Yiyun; Li, Xiuxia; Yang, Kehu | 2023 | Journal of Science and Medicine in Sport | 26 | 4-5 | 232-240 |
| Umbrella review of musculoskeletal injury burden in dancers: implication for practice and research | Anand Prakash, Akilesh; K, Mahesh; Akilesh, Vinitha | 2024 | The Physician and Sportsmedicine | 52 | 1 |  |
| Tools and guidelines to assess the appropriateness of medication and aid deprescribing: An umbrella review | Anlay, Degefaye Zelalem; Paque, Kristel; Van Leeuwen, Ellen; Cohen, Joachim; Dilles, Tinne | 2024 | British journal of clinical pharmacology | 90 | 1 | 12-106 |
| Treatment options for oropharyngeal carcinoma - An umbrella review | Plutecki, Dawid; Szczepanek, Elżbieta; Szczepanek, Małgorzata; Nasser, Ameen; Hajdyła, Paweł; Wrona, Andrzej | 2023 | Folia Medica Cracoviensia | 63 | 3 | 103-124 |
| Autoimmune diseases and adverse pregnancy outcomes: an umbrella review | Singh, Megha; Wambua, Steven; Lee, Siang Ing; Okoth, Kelvin; Wang, Zhaonan; Fazla, Fathima; Fayaz, Ahamed; Eastwood, Kelly-Ann; Nelson-Piercy, Catherine; Nirantharakumar, Krishnarajah | 2023 | The Lancet | 402 |  | S84 |
| The Relationship between metformin consumption and cancer risk: an updated umbrella review of systematic reviews and meta-analyses | Najafi, Farid; Rajati, Fatemeh; Sarokhani, Diana; Bavandpour, Maryam; Moradinazar, Mehdi | 2023 | International Journal of Preventive Medicine | 14 | 1 | 90 |
| Effectiveness and safety of awake prone positioning in COVID-19-related acute hypoxaemic respiratory failure: an overview of systematic reviews | Li, Ya; Zhao, Guixiang; Ma, Yizhao; Wang, Lu; Liu, Ying; Zhang, Hailong | 2024 | BMC Pulmonary Medicine | 24 | 1 | 5 |
| Associations between the use of red yeast rice preparations and adverse health outcomes: An umbrella review of meta-analyses of randomized controlled trials | Ma, Zhen-yu; Yang, Shu-ping; Li, Ying; Xu, Tian-tian; Yang, Ya-lin; Yang, Hui-yong; Li, Heng-bing; Zhou, Le-jin; Diao, Yong; Li, Su-yun | 2024 | Journal of Integrative Medicine | 22 | 2 | 126-136 |
| Overview of Cochrane systematic reviews for rehabilitation interventions in individuals with cerebral palsy: A mapping synthesis | Liguori, Sara; Young, Vanessa M; Arienti, Chiara; Pollini, Elisa; Patrini, Michele; Gimigliano, Francesca; Negrini, Stefano; Kiekens, Carlotte | 2023 | Developmental Medicine & Child Neurology | 65 | 10 | 1280-1291 |
| State of the art of the literature on definitions of self-criticism: a meta-review | Zaccari, Vittoria; Mancini, Francesco; Rogier, Guyonne | 2024 | Frontiers in Psychiatry | 15 |  |  |
| Effectiveness of tranexamic acid in orthognathic surgery: a systematic review of systematic reviews | AlQahtani, Feras AlMofreh; Kuriadom, Sam Thomas; Varma, Sudhir; AlAnzy, Hamedy; AlOtaibi, Sami | 2023 | Journal of stomatology, oral and maxillofacial surgery | 124 | 6(suppl2) |  |
| The effects of pro-, pre-, and synbiotics supplementation on polycystic ovary syndrome: an umbrella review of meta-analyses of randomized controlled trials | Talebi, Sepide; Zeraattalab-Motlagh, Sheida; Jalilpiran, Yahya; Payandeh, Nastaran; Ansari, Shakila; Mohammadi, Hamed; Djafarian, Kurosh; Ranjbar, Mahsa; Sadeghi, Sara; Taghizadeh, Mahdiyeh | 2023 | Frontiers in Nutrition | 10 |  |  |
| Effects of turmeric (Curcuma longa) supplementation on glucose metabolism in diabetes mellitus and metabolic syndrome: An umbrella review and updated meta-analysis | Pathomwichaiwat, Thanika; Jinatongthai, Peerawat; Prommasut, Napattaoon; Ampornwong, Kanyarat; Rattanavipanon, Wipharak; Nathisuwan, Surakit; Thakkinstian, Ammarin | 2023 | PloS One | 18 | 7 | e0288997 |
| Association between the risk of preterm birth and low birth weight with periodontal disease in pregnant women: an umbrella review | Padilla-Cáceres Tania; Arbildo-Vega, Heber Isac; Caballero-Apaza, Luz; Cruzado-Oliva, Fredy; Mamani-Cori, Vilma; Cervantes-Alagón, Sheyla; Munayco-Pantoja, Evelyn; Panda, Saurav; Vásquez-Rodrigo, Hernán; Castro-Mejía, Percy | 2023 | Dentistry Journal | 11 | 3 | 74 |
| Efficacy and safety of ketamine and esketamine for unipolar and bipolar depression: an overview of systematic reviews with meta-analysis | Rodolico, Alessandro; Cutrufelli, Pierfelice; Di Francesco, Antonio; Aguglia, Andrea; Catania, Gaetano; Concerto, Carmen; Cuomo, Alessandro; Fagiolini, Andrea; Lanza, Giuseppe; Mineo, Ludovico | 2024 | Frontiers in psychiatry | 15 |  | 1E+06 |
| The Association of Vitamin D with Non-Melanoma Skin Cancer Risk: An Umbrella Review of Systematic Reviews and Meta-Analyses | Seretis, Konstantinos; Bounas, Nikolaos; Sioka, Chrissa | 2023 | Medicina | 59 | 12 | 2130 |
| Complementary and alternative treatments for insomnia disorder: a systematic umbrella review | Ell, Johanna; Schmid, Sarah R; Benz, Fee; Spille, Lukas | 2023 | Journal of sleep research | 32 | 6 | e13979 |
| Child abuse and neglect during the COVID-19 pandemic: an umbrella review | Carsley, Sarah; Thomas, Sera; Oei, Tiffany; Smith, Brendan; Harrington, Daniel; Pike, Ian; Macpherson, Alison K; Richmond, Sarah A | 2024 | Child Abuse & Neglect | 149 |  |  |
| Umbrella review: Newly graduated nurses' experiences of providing direct care in hospital settings | Kaldal, Maiken Holm; Conroy, Tiffany; Feo, Rebecca; Grønkjaer, Mette; Voldbjerg, Siri Lygum | 2023 | Journal of Advanced Nursing | 79 | 6 | 2058-2069 |
| Antenatal, Intrapartum and Postpartum Interventions for Preventing Postpartum Urinary and Faecal Incontinence: An Umbrella Overview of Cochrane Systematic Reviews | Sananès, Juliette; Pire, Sophie; Feki, Anis; Boulvain, Michel; Faltin, Daniel L | 2023 | Journal of clinical medicine | 12 | 18 | 6037 |
| The prevalence of pain in chronic diseases: an umbrella review of systematic reviews | Viderman, Dmitriy; Tapinova, Karina; Aubakirova, Mina; Abdildin, Yerkin G | 2023 | Journal of Clinical Medicine | 12 | 23 | 7302 |
| Association between ultra-processed foods consumption and the risk of hypertension: an umbrella review of systematic reviews | Wang, Ziyi; Lu, Cuncun; Wang, Yongsheng; Fenfen, E; Mentis, Alexios Fotios A; Li, Xiuxia; Yang, Kehu | 2023 | Hellenic Journal of Cardiology | 76 |  | 99-109 |
| Maternal health outcomes associated with ambient air pollution: An umbrella review of systematic reviews and meta-analyses | Mazumder, Hoimonty; Rimu, Fariha Hoque; Shimul, Monir Hossain; Das, Jyoti; Gain, Easter Protiva; Liaw, Winston; Hossain, M. Mahbub | 2024 | Science of The Total Environment | 914 |  |  |
| An umbrella review of the evidence to guide decision-making in acupuncture therapies for chemotherapy-induced peripheral neuropathy | Shi, Hongshuo; Yuan, Xin; Fan, Weijing; Yang, Xiao; Liu, Guobin | 2023 | Journal of Cancer Research and Clinical Oncology | 149 | 17 | 15939-15955 |
| A Systematic Review of Systematic Reviews on the Use of Aromatase Inhibitors for the Treatment of Endometriosis: The Evidence to Date | Peitsidis, Panagiotis; Tsikouras, Panagiotis; Laganà, Antonio Simone; Laios, Alexandros; Gkegkes Ioannis D; Iavazzo, Christos | 2023 | Drug Design, Development and Therapy | 17 |  | 1329-1346 |
| Rehabilitation Interventions for Complex Regional Pain Syndrome: An Overview of Systematic Reviews | Shafiee, Erfan; MacDermid, Joy; Packham, Tara; Grewal, Ruby; Farzad, Maryam; Bobos, Pavlos; Walton, David | 2023 | The Clinical Journal of Pain | 39 | 9 | 473-483 |
| Transitional care model for older adults with multiple chronic conditions: An evaluation of benefits utilising an umbrella review | Berthelsen, Connie; MÃ¸ller, Nicoline; Bunkenborg, Gitte | 2024 | Journal of Clinical Nursing | 33 | 2 | 481-496 |
| Self-management strategies for people with epilepsy: An overview of reviews | Goh, Sarah L.; Harding, Katherine E.; Lewis, Annie K.; Taylor, Nicholas F.; Carney, Patrick W. | 2024 | Epilepsy & Behavior | 150 |  | 1E+05 |
| Medication Adherence Among Patients With Kidney Disease: An Umbrella Review | Tesfaye, Wubshet; Parrish, Nicholas; Sud, Kamal; Grandinetti, Amanda; Castelino, Ronald | 2024 | Advances in Kidney Disease and Health | 31 | 1 | 68-83 |
| Does valerian work for insomnia? An umbrella review of the evidence | Valente, Valéria; Machado, Daniela; Jorge, Susana; Drake, Christopher L.; Marques, Daniel Ruivo | 2024 | European Neuropsychopharmacology | 82 |  |  |
| Probiotics for functional constipation in children: an overview of overlapping systematic reviews | Zhang, Yunxin; Li, Aiping; Qiu, Jing; Wen, Hua; Zhang, Hanwen; Sun, Xiangjuan | 2024 | Frontiers in Cellular and Infection Microbiology | 13 |  |  |
| Immunogenicity and safety of inactivated quadrivalent influenza vaccine compared with the trivalent vaccine for influenza infection: an overview of systematic reviews | Carregaro, Rodrigo Luiz; Roscani, Alessandra N. C. P.; Raimundo, Augusto Cesar Sousa; Ferreira, Larissa; Vanni, Tazio; da Graça Salomão, Maria; Probst, Livia Fernandes; Viscondi, Juliana Yukari K. | 2023 | BMC Infectious Diseases | 23 | 1 | 563 |
| A critical overview of systematic reviews and meta-analyses of extracorporeal shockwave therapy for knee osteoarthritis | Zhou, Qinxin; Chen, Jixin | 2024 | Asian Journal of Surgery | 47 | 7 | 2975-2984 |
